# Feasibility study of short hydration using oral rehydration solution in cisplatin including chemotherapy of lung cancer

**DOI:** 10.1186/s40780-016-0041-z

**Published:** 2016-03-05

**Authors:** Junya Sato, Naoto Morikawa, Hiroo Nitanai, Hiromi Nagashima, Satoru Nihei, Kohei Yamauti, Kenzo Kudo

**Affiliations:** Department of pharmacy, Iwate Medical University Hospital, 19-1Uchimaru, Morioka, Iwate 020-8505 Japan; Department of Clinical Pharmaceutics, School of Pharmacy, Iwate Medical University, 2-1-1 Nishitokuta, Yahaba, Iwate 028-3694 Japan; Department of Internal Medicine, Division of Pulmonary Medicine, Allergy and Rheumatology, School of Medicine, Iwate Medical University, 19-1Uchimaru, Morioka, Iwate 020-8505 Japan

**Keywords:** Cisplatin, Oral rehydration solution, Chemotherapy, Renal dysfunction, Short hydration

## Abstract

**Background:**

Cisplatin (CDDP) is used as a key anticancer drug for solid cancers, including lung cancer. However, a large quantity of fluid replacement is required to prevent renal dysfunction. This requirement have made outpatient chemotherapies including CDDP administration less popular among the available therapeutic options. We designed a short-term hydration regimen combined with oral rehydration solution (ORS) that has a supplementary water ability equivalent to intravenous electrolyte maintenance infusion and investigated its safety and feasibility in the CDDP including chemotherapy.

**Methods:**

The subjects received chemotherapy including CDDP administration (60–80 mg/m^2^) for untreated lung cancer were recruited. The intravenous hydration was infused at around 2000 mL on Day 1, and patients drank ORS at a dose of 1000 mL/day for 3 days. Any renal dysfunction, gastrointestinal symptoms or other tolerability variables pertaining to the remaining three cycles of this regimen were analyzed in the patients who were able to continue treatment after the second cycle.

**Results:**

The majority (29/35, 82.9 %) of patients completed intake of ORS for 3 days. The mean ± standard deviation of patient body-surface area-adjusted estimated glomerular filtration rate (eGFR), serum creatinine (sCre) and urea nitrogen from the initial therapy to 1 month after the last administration changed from 79.8 ± 11.7–67.0 ± 16.9 mL/min (*p* = 0.15), 0.70 ± 0.13–0.85 ± 0.27 mg/dL (*p* = 0.02), and 14.3 ± 3.8–17.1 ± 5.4 mg/dL (*p* = 0.09), respectively. The CTCAE ver 4.0 grades 1 or 2 adverse events pertaining to renal function after the last administration were 2 (5.7 %)/2 (5.7 %) patients assessed by sCre, and 14 (40.0 %)/12 (34.3 %) patients assessed by eGFR, respectively. There was no patient with ≥3 grade renal dysfunction based on either evaluation.

**Conclusions:**

Based on the results of this study, supplementary use of the ORS as a method of short-term hydration may be a feasible regimen for shortening infusion times and improving safety for those undergoing chemotherapy including CDDP administration.

## Background

Recently, cancer chemotherapy in the outpatient setting has become more popular as it may improve the quality of life of cancer patients. The increased use of this treatment option has corresponded to the increase in its demand in cancer patients. However, outpatient chemotherapy, including administration of cisplatin (CDDP), a key anti-cancer drug, in lung cancer patients undergoing chemotherapy has not been a popular treatment option in Japan due to its requirement for large quantities of hydration over long durations to prevent renal dysfunction. In contrast, most other foreign countries have adopted short-term duration hydration regimens involving a minimum of approximately 2000 mL. However, CDDP administration using short-term hydration regimens has been associated with renal dysfunction in some cases and may lead to treatment discontinuation [1]. Therefore, it is important to further investigate safety improvements to be considered when using CDDP administration and short-term hydration.

An oral rehydration solution (ORS) with a water-supplementing ability equivalent to that of intravenous electrolyte maintenance infusion was available to address dehydration due to a variety of conditions, including diarrhea. The pharmacologic effect of ORS is based on the cotransport of sodium and glucose in the small intestine. This results in effective water absorption based on the physiological movement of sodium during this cotransporting process [2]. ORS is characterized by an abundance of electrolytes and lower levels of glucides than would be found in similar sports drinks. The effectiveness and safety of ORS in dehydration and in postoperative recovery have been confirmed and demonstrated equality with an intravenous maintenance infusion medium in humans [3, 4]. However, there have been no reports regarding the application of ORS as a form of oral hydration to facilitate renal drug excretion. We hypothesized that application of ORS in addition to chemotherapy, including CDDP administration, in outpatients may shorten infusion times and allow for safety improvement when ORS was used as a substitute for intravenous hydration, or added as a supplement to intravenous hydration. Regarding the possibility that ORS was more effective than normal oral water intake in the hydration accompanying CDDP administration, we performed our primary examination using animal models. Our results indicated that ORS drinking reduced renal dysfunction as compared with normal oral water in a CDDP-induced renal dysfunction rat model [5].

The current study was designed to assess the feasibility of using ORS in patients receiving chemotherapy, including CDDP administration, based on the results of our previous studies.

## Methods

### Subjects

Subjects in this study who had received an initial diagnosis of lung cancer and elected to receive chemotherapy, including CDDP administration, at the Iwate Medical University hospital from May 2013 to December 2014 were recruited. The study entry conditions included normal renal function (creatinine clearance, ≥50 mL/min), availability for administration of CDDP, capability to adhere to dietary constraints and orally consume ≥1000 mL of water per day, performance status (PS) of 0 or 1, written agreement to enter the study, and age ≥18 years. The analysis of the study data was performed for patients who met all entry conditions and tolerated the first cycle of CDDP administration. The evaluation period was 1 month after up to four cycles.

### Treatment schedule

The treatment schedule including hydration is displayed in Table 1. The chemotherapy regimen involved 80 mg/m^2^ of CDDP administrated every 3 weeks with gemcitabine hydrochloride (GEM: 1000 mg/m^2^, Day 1, 8) or docetaxel (DTX: 60 mg/m^2^, Day 1) for patients with non-small cell lung cancer (NSCLC), pemetrexed sodium hydrate (PEM: 500 mg/m^2^, Day 1) for NSCLC or malignant pleural mesothelioma, and etoposide (VP-16: 100 mg/m^2^, Day 1–3) for patients with small-cell lung cancer. An irinotecan hydrochloride (CPT-11: 60 mg/m^2^, Day 1, 8) was administrated every 3 weeks with 60 mg/m^2^ of CDDP to patients with small-cell lung cancer. The total intravenous hydration volume associated with CDDP administration on Day 1 was 1700–2150 mL. Additionally, 600 mL of intravenous hydration 2 days afterward was administered to patients on the CDDP-VP-16 combination therapy. ORS (OS-1, Otsuka Pharmaceutical Factory Inc; Tokushima, Japan) was administrated at 1000 mL per day from the infusion start date for 3 days as oral hydration. The antiemetic therapy prior to CDDP infusion was administered using 125 mg of aprepitant orally and 0.75 mg of palonosetron hydrochloride, coupled with 9.9 mg of dexamethasone sodium phosphate administered intravenously on Day 1. Additionally, 80 mg of aprepitant and 8 mg of dexamethasone were administered orally 2 days afterwards. In those receiving CDDP-VP-16 therapy, 9.9 mg of dexamethasone was administered with VP-16 intravenously on Day 2 and 3. The first cycle of chemotherapy was performed in hospital, and subsequent chemotherapy was largely ambulatory.

**Table 1:** CDDP administration regimen by the short hydration

Rp	Drugs	Volume	Infusion time
1	ORS	1000 mL	Oral intake
2	Aprepitant (125 mg)		Oral intake
3	Palonosetron (0.75 mg) Dexamethasone (9.9 mg) diluted with Saline	100 mL	30 min
4	Maintenance solution mixed MgSO4 (8 mEq)	500 mL	60 min
5	VP-16, CPT-11, GEM, DTX, PEM diluted with Saline or 5%Glusose^a^	100 ~ 500 mL	10 ~ 90 min
6	Cisplatin 60 ~ 80 mg/m2 diluted with Saline	500 mL	120 min
7	Flosemide (20 mg) diluted with Saline	50 mL	5 min
8	Mentenance solution mixed MgSO4 (8 mEq)	500 mL	60 min

^a^ VP-16 (100 mg/m2)diluted with saline 500 mL; 90 min, CPT-11 (60 mg/m2) diluted with saline 500 mL; 90 min

GEM (1000 mg/m2) diluted with 5%glucose; 30 min, DTX (60 mg/m2) diluted with saline 250 mL; 60 min

PEM (500 mg/m2) diluted with saline 100 mL; 10 min

### Primary endpoint

As the primary endpoint, we evaluated renal function through levels of serum creatinine (sCre), urea nitrogen (BUN) and the Japanese body-surface area-adjusted estimated glomerular filtration rate (eGFR) [6]. These values were assessed just before the administration of each cycle and 1 month after the last administration. Changes in sCre levels and normal physique eGFR (mL/min/1.73 m^2^) were evaluated using CTCAE ver 4.0. sCre was evaluated as grade 0 (<1.5 times), 1 (≥1.5– < 2.0 times), 2 (≥2.0– < 3.0 times), or 3 (≥3.0 times) as compared with a baseline measurement made just before administration of the first cycle. eGFR was evaluated as grade 0 (≥80 mL/min/1.73 m^2^), 1 (<80– ≥ 60 mL/min/1.73 m^2^), 2 (<60– ≥ 30 mL/min/1.73 m^2^), or 3 (<30– ≥ 15 mL/min/1.73 m^2^).

### Secondary endpoint

As secondary endpoints, we evaluated nausea, vomiting, dietary intake, completion rate and discomfort following 3 days ORS intake during the first cycle. Vomiting episode and nausea were assessed using a six grade nausea scale (0 = no nausea, 5 = severe nausea). A diet intake ratio was used to assess the quantity of average dietary intake as compared with before the initiation of therapy, and was evaluated as a gastrointestinal symptom. Discomfort from 3 days of ORS intake was evaluated using a six grade scale (0 = no discomfort, 5; = severe discomfort). These endpoints were self-reported by patients in writing every 24 h for 5 days from the initiation of CDDP administration.

### Statistics and ethical evaluation

The comparisons between the numerical values for sCre, BUN and eGFR collected before (control) and after study drug administration were performed as multiple comparisons using Dunnett’s t-tests. A bilateral hazard ratio less than 5 % was determined to represent a significant difference. This study was conducted with the permission (H23-58) of the Iwate Medical University School of Medicine Ethical Review Board.

## Results

### Patient background

**Fig. 1:**
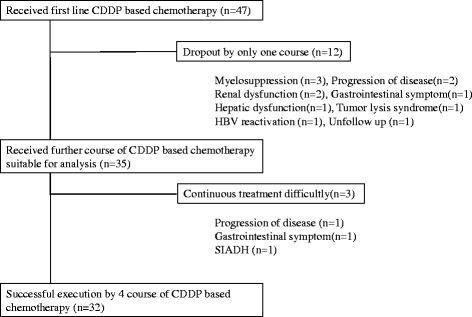
Consort diagram of this study. Figure 1 indicated the consort diagram of this study. Forty seven patient agreed to the participation in study and received first line CDDP based chemotherapy. Twelve patients had difficulty in treatment continuously at the first cycle by illustrated reasons. Thirty five patients received further course of CDDP based chemotherapy and suitable for analysis

A consort diagram of this study is displayed in Fig. 1. Forty seven patients who started chemotherapy, including CDDP administration, for untreated lung cancer entered into this study. Twelve patients (25.5 %) discontinued chemotherapy, including CDDP administration, within the first cycle. The reasons given for discontinuation during the first cycle were myelosuppression (*n* = 3), progression of disease (*n* = 2), impaired renal function (*n* = 2), severe gastrointestinal symptoms (*n* = 1), hepatic dysfunction (*n* = 1), tumor lysis syndrome (*n* = 1), hepatitis B virus reactivation (*n* = 1), and loss to follow-up (*n* = 1). An analysis of outcomes was performed for the 35 patients who could continue chemotherapy, including CDDP administration, in subsequent cycles.

The characteristics of these 35 patients are displayed in Table 2. Among the 35 patients who continued to undergo subsequent cycles of chemotherapy, three could not complete the forth cycle due to disease progression (*n* = 1), syndrome of inappropriate secretion of antidiuretic hormone (*n* = 1), and refusal based on treatment-resistant vomiting (*n* = 1).

**Table 2:** Patient characteristics

Patient characteristics		*n* = 35
Sex		male;28 : female;7 (80 %:20 %)
Age		65.7 ± 5.7
Performance status (ECOG)	0	3 (8.6 %)
	1	32 (91.4 %)
Histology	Small cell carcinoma	17 (48.6 %)
	Adenocarcinoma	12 (34.3 %)^a^
	Squamous carcinoma	3 (8.6 %)
	Pleural mesothelioma	2 (5.7 %)
	Large cell carcinoma	1 (2.9 %)
Clinical stage (UICC TNM)	I B	1 (2.9 %)
	IIA	2 (5.7 %)
	IIB	1 (2.9 %)
	IIIA	6 (17.1 %)
	IIIB	3 (8.6 %)
	IV	21 (60.0 %)
	Unknown	1 (2.9 %)
Smoking status	Never	6 (17.1 %)
	Ever	11 (31.4 %)
	Current	18 (51.4 %)
CDDP dosage (mg/m2)		69.7 ± 8.9
CDDP cumulative dosage (mg/m2)		278.6 ± 63.3
Course		4.1 ± 0.9
CDDP dose reduction (%)	None	21 (60 %)
	1 stage reduction	8 (23 %)
	2 stage reduction	6 (17 %)
Regimen	CDDP + MTA ± BV	12 (34 %)
	CDDP + VP-16	12 (34 %)
	CDDP + CPT-11	5 (14 %)
	CDDP + GEM	5 (14 %)
	CDDP + DTX	1 (4 %)
	Hydration volume (mL)	1937 ± 193
	Infusion time (hr)	4.8 ± 1.1
Renal function	sCre (mg/dL)	0.7 ± 0.1
	BUN (mg/dL)	14.3 ± 3.8
	Adjusted eGFR (mL/min)	79.8 ± 9.9

mean ± SD

^a^ Driver mutation status; Epidermal Growth Factor Receptor; EGFR (+); *n* = 2, Anaplastic lymphoma kinase; ALK (+); *n* = 1

### Change in the renal function

**Fig. 2:**
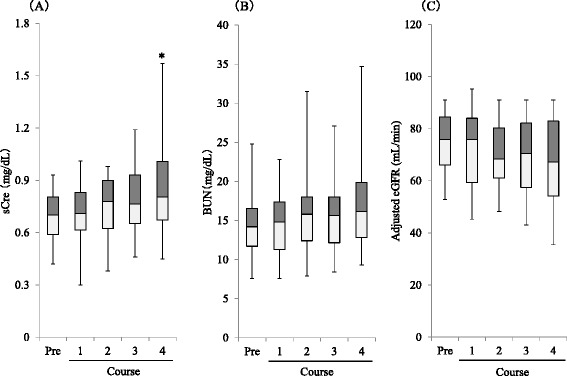
Boxplot of renal functions in the patient received CDDP based chemotherapy. Figure 2 indicated change in the renal functions in the 35 patients. **a** Left graph indicated boxplot of sCre (mg/dL). **b** Central graph indicated boxplot of urea nitrogen (mg/dL). **c** Right graph indicated boxplot of body-surface area-adjusted eGFR (mL/min). An asterisk (*) indicated statistically significant difference less than 5 % of hazard ratios by dunnett *t*-test

**Fig. 3:**
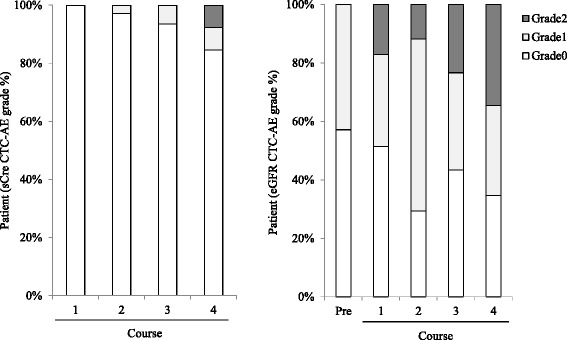
Renal dysfunction evaluated by CTC-AE. Figure 3 indicated the renal dysfunction evaluated by CTC-AE in the 35 patients. Left graph indicated grade distribution evaluated by sCre elevation (Grade 0; base-line × <1.5times, Grade 1; ≧1.5– < 2 times, Grade 2; ≧2– < 3 times). Right graph indicated grade distribution evaluated by eGFR decrease (Grade 0; ≧80 mL/min/1.73 m^2^, Grade 1; <80– ≧60 mL/min/1.73 m^2^, Grade 2; <60– ≧30 mL/min/1.73 m^2^)

Changes in patient renal function (sCre and BUN levels, body-surface area-adjusted eGFR) in the 35 analyzed patients are represented as box plots in Fig. 2. As compared with numerical average values collected before study drug the administration, only sCre levels after 4 cycles increased significantly (*p* < 0.05). The renal function evaluations by CTCAE ver 4.0 are displayed in Fig. 3. The sCre grade during the chemotherapy cycle was evaluated as grade 0 (no change) for 33 patients (94 %), and as grade 1 in two patients (6 %). The sCre grade after the last administration was evaluated as grade 0 for 31 patients (88.6 %), grade 1 for two patients (5.7 %), and grade 2 for two patients (5.7 %). Regarding eGFR, 15 (42.9 %) patients were evaluated as grade 1 prior to study drug administration. The change in the eGFR during the chemotherapy cycle was evaluated as grade 0 (no change) in seven patients (20 %), grade +1 increase in 23 patients (65.7 %), and grade + 2 increase in five patients (14.3 %). The eGFR grade after the last administration was evaluated as grade 0 in nine patients (25.7 %), grade 1 in 14 patients (40.0 %), an grade 2 in 12 patients (34.3 %). Five patients with either sCre or eGFR increases of more than grade + 2 completed 4 cycle of planned chemotherapy. Three of these patients improved these grades shortly after chemotherapy completion.

### Gastrointestinal symptom and drinking evaluation of ORS

**Fig. 4:**
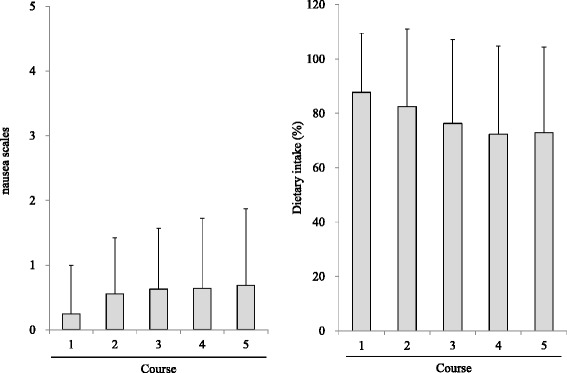
Nausea scale and dietary intake (%) evaluated by patient self-writing scale (*n* = 35). Fig. 4 indicated gastrointestinal symptom evaluated by patient self-writing scale in the 35 patients. Left graph indicated nausea scale (o;no nausea, 5; severe nausea). Right graph indicated dietary intake (%) compared with the usual food consumption before the treatment

The nausea scale and diet intake ratio are represented in Fig. 4. The nausea scale evaluated using a six stage patient self-report scale showed an average range of 0.2–0.7 for 5 days. Of the 35 patients, vomiting during the first cycle did not occur on Day1 or 2, but did occur on Day 3 in one patient (1 time), on Day 4 in one patient (3 times), and on Day 5 in one patient (2 times). Average dietary intake was maintained at approximately 72.9–87.7 % as compared with the ratio calculated before chemotherapy for 5 days. The six stage discomfort scale of ORS drinking for 3 days was increased over days by average of 0.5 on Day 1, 0.9 on Day 2 and by 1.3 on Day 3. Overall, 29 patients (82.8 %) were able to complete drinking for 3 days.

## Discussion

It was also determined that renal dysfunction could occur at high frequencies (28–36 %) when including the slight impairments caused by ≥50 mg/m^2^ of CDDP administration, even when hydration and diuresis were conducted adequately [6, 7]. The renal dysfunction caused by CDDP was attributed to accumulation of CDDP in renal tubules and associated tubular cell necrosis [8]. Hydration reduces renal dysfunction by diluting the urinary CDDP concentration, as well as shortening the contact time in the renal tubule by early excretion. In particular, the efficient dilution and excretion attributable to the hydration of the CDDP free-form represented much of the early excretion (within 2 h after administration), which was considered to be significant, as the form of CDDP not bound to proteins in the blood has been implicated in glomerular toxicity [9]. Preservation of the early urine flow by the hydration seems to be required for prevention of renal dysfunction during CDDP administration. Although hydration seemed beneficial in general, there was some concern over whether renal dysfunction could be even further reduced if the hydration increased the volume. The results of one study suggested that increasing hydration volumes had no influence on the incidence of renal dysfunction by showing no differences when hydration volumes during CDDP administration were more than 2000 mL or not [10]. Some CDDP administration regimens with infusion volumes of approximately 2000 mL have been termed short-term hydration, and have been investigated vigorously. Tiseo et al. were the first to report the general idea of short-term hydration, suggesting a total infusion volume of 2100 mL for 4.25 h in addition to chemotherapy including ≥75 mg/m^2^ of CDDP in lung cancer patients [1]. Five (4.6 %) of the 107 patients discontinued treatment due to renal dysfunction. However, the definition of short-term hydration was not clear, and wide variations in variables, including hydration volume, composition of the infusion, addition of magnesium, rank order and the use of diuretics, were observed in subsequent studies. Additionally, safety results from short-term hydration experiments performed in Japanese prospective studies generally showed that grade ≥2 renal dysfunction was detected at a rate of less than 10 % [11, 12]. Therefore, we had designed a regimen including the combined use of ORS with existing short-term hydration for the purpose of improving the safety issues associated with renal dysfunction over prolonged infusion times.

Renal dysfunction, as evaluated by the sCre, was only observed to occur in two patients and did not exceed grade 2 in any patient using our method combining ORS with an intravenous infusion of an average of 1930 mL (4.7 h). On the other hand, there were more patients (34.3 %) with grade 2 renal dysfunction after their last CDDP administration as evaluated by eGFR when compared with sCre; however, this was not an unexpected finding, as there were already 15 patients (42.9 %) with decreased renal function equal to grade 1 before the first cycle. Importantly, most of the evaluated patients did not develop severe renal dysfunction (grade ≧3), and completed all four scheduled chemotherapy cycles. The outcomes for the patients showing grade 2 renal dysfunction did not exhibit further progression, and some of these patient even showed signs of recovery. The antiemetic effect of this treatment seemed to be good based on the nausea grade, the number of vomiting instances, and lack of effects on dietary intake. The high compliance and tolerability of ORS drinking were confirmed, as all patients were available for 1000 mL of ORS drinking on the first day. Side effects possibly attributable to ORS drinking were only slightly observed as abdominal distension, nausea and diarrhea. It should be noted, however, that these symptoms were considered as a side effect of the chemotherapy.

The safety of this regimen considered as follows. Nine of 47 patients who received initial chemotherapy were not able to receive second cycle for a treatment-related side effect. Also, reduction of CDDP was required for 40 % of patients after the second cycle. Most of reasons were due to myelosuppression. The side effects such as the myelosuppression occurred even if short hydration supplemented with ORS was used. The initial chemotherapy should be carried out in hospitalization, and adequate attention seemed to be required for side effects such as the myelosuppression in the subsequent chemotherapy. It should also be noted, however, that some patients with renal dysfunction up to grade 2 were observed even when ORS was used as a supplement to intravenous transfusion in this study. Although our results were suggestive of the safety of this regimen, the study was not sufficiently powered to conclude that supplementary use of the ORS was able to relieve renal dysfunction due to CDDP. The mean age of the study participants was comparatively high (65.7 years), and the 42.9 % of the patients with <80 mL/min in eGFR at study entry may have influenced the study results. However, improving the safety of CDDP for these patient groups remains an important goal for those in clinical practice. Therefore, a modification of the hydration regimen was thought to be necessary in order to further reduce the renal dysfunction associated with the use of CDDP. The use of diuretics, such as mannitol or furosemide [13] and Na, Cl rich infusion [14, 15], as well as magnesium supplementation [16], have all been demonstrated as important for the prevention of renal dysfunction due to CDDP. In this study, we used furosemide due to its rapid diuretic effect; however, it was not administered before CDDP administration. It may be the case that use of furosemide before CDDP administration may produce an earlier diuretic effect. Additionally, maintenance infusion was used for hydration, and its Na and Cl quantity was 25 % saline. It may have been more useful to employ higher amounts of saline for hydration. We consider the factor which might influence the renal function of the patients as follows. Patients with hypoalbuminemia in particular have been shown to experience renal dysfunction rather easily as a result of CDDP treatment [10]. It should be noted, however, that hypoalbuminemia < 3.0 g/dL was not detected in the patient with sCre elevation of grade 2 in this study. Also, the poor PS might influence renal function. However, the patients who received CDDP were all PS >1 in this study. Therefore, it was thought that there was little effect of the PS on the results. Combination use of nonsteroidal antiinflammatory drug (NSAIDs) was considered as the factor which influenced renal dysfunction [17, 18]. Four patients used NSAIDs during duration of chemotherapy routinely. Three of four patients had a grade aggravation evaluated by eGFR. Therefore, combination use of NSAIDs might influence renal function in these patients. Renal function as evaluated by sCre may result in underestimations because the biosynthesis of creatinine decreases with the muscle degradation that accompanies the aging process (the so-called creatinine blind). We used sCre, eGFR and BUN as parameter of renal function in this study to investigate the complementarity of these results.

Oral hydration using ORS as a substitute for water was thought to be useful on the initial CDDP administration day for at least two reasons. First, hydration is possible before an infusion begins and can be administered anywhere. Secondly, the excretion of the CDDP protein-free form, which exists at its highest levels in the early stage of CDDP administration, was promoted pharmacologically due to its rapid water absorption. ORS remained useful during the remainder of the CDDP regimen because it was available for hydration in a non-intravenous manner, highly accessible in drug stores without a prescription, low in cost and safe with rare side effects, including diarrhea.

## Conclusions

We demonstrated the potential of short-term hydration using ORS as a well-tolerated, safe tool to supplement intravenous hydration during CDDP administration. Reconsideration of current hydration regimens, as well as one or more noninferiority studies with or without ORS, seems necessary in light of our study findings.

## Abbreviations

BUN: urea nitrogen
CDDP: cisplatin
CPT-11: irinotecan hydrochloride
CTCAE: common terminology criteria for adverse events
DTX: docetaxel
eGFR: estimated glomerular filtration rate
GEM: gemcitabine hydrochloride
ORS: oral rehydration solution
PEM: pemetrexed sodium hydrate
sCre: serum creatinine
VP-16: etoposide
